# Sustainable Utilisation and Management of Food Waste for High-Value Products

**DOI:** 10.3390/foods12152872

**Published:** 2023-07-28

**Authors:** Amit K. Jaiswal

**Affiliations:** 1 School of Food Science and Environmental Health, College of Sciences and Health, Technological University Dublin—City Campus, Central Quad, Grangegorman, D07 ADY7 Dublin, Ireland; amit.jaiswal@TUDublin.ie; 2 Environmental Sustainability and Health Institute (ESHI), Technological University Dublin—City Campus, Grangegorman, D07 H6K8 Dublin, Ireland

Welcome to the Special Issue on “Sustainable Utilisation and Management of Food Waste for High-Value Products”. This Special Issue focuses on one of the most critical challenges facing our world today—the efficient management and utilisation of food waste. It presents a variety of scientific investigations from renowned scholars and researchers worldwide to address this challenge, covering an array of topics ranging from fundamental research to real-world applications.

Food waste is a global dilemma that requires urgent attention. According to the Food and Agriculture Organization (FAO), approximately one-third of food produced for human consumption worldwide, or around 1.3 billion tons, is wasted annually [1]. This enormous waste entails significant economic, environmental, and food security implications. Economically, the worldwide financial cost of food wastage exceeds USD 1 trillion annually [2]. Environmentally, if wasted food were considered a country, it would be the third-largest emitter of greenhouse gases. Additionally, the disposal of wasted food exacerbates landfill pressures and depletes limited natural resources used in food production, such as land and water. From a food security perspective, the shocking extent of food waste simply contrasts with the reality of almost 720 and 811 million people suffering from hunger globally [3].

However, as the proverb goes, “every cloud has a silver lining”, and the food waste problem is no exception. The untapped potential exists in food waste to be exploited for generating high-value products [4]. Using innovative technologies and sustainable strategies, food waste can be transformed into a variety of products, from biofuels, fertilisers, and feedstocks to functional food ingredients and packaging materials [4]. The extraction of useful compounds, such as dietary fibres, phenolics, and proteins, from food waste opens doors to an array of applications in the food, pharmaceutical, and cosmetic industries. By promoting the concept of a circular economy, this Special Issue aims to highlight research efforts focusing on the sustainable utilisation and management of food waste, transforming this global problem into a treasure trove of opportunities. A total of eight notable papers are published in this Special Issue.

The research article, entitled “Crude Pectic Oligosaccharide Recovery from Thai Chok Anan Mango Peel Using Pectinolytic Enzyme Hydrolysis”, by Wongkaew et al. [5] investigates the potential of mango peel as a source of pectic oligosaccharides for probiotic growth and prebiotic activity. The authors determined the optimal conditions for enzyme hydrolysis of mango peel pectin and observed how these conditions affect two bacterial species, *Lactobacillus reuteri* and *Bifidobacterium animalis*. The results showed a high prebiotic activity and the production of short-chain fatty acids, which are beneficial for gut health. This study suggests that mango peel-derived pectic oligosaccharides can serve as a sustainable, beneficial by-product of the fruit industry.

The research article, entitled “Physical Properties of Flours Obtained from Wasted Bread Crusts and Crumbs”, by Juan Fernández-Peláez et al. [6] examines the potential of wasted bread, one of the most commonly discarded foods, as a sustainable source of flour. The researchers analysed flours derived from the crumbs and crusts of eight different breads to understand the influence of bread type and its parts (crumb or crust) on the characteristics of flour. It was observed that bread flours possess higher water-holding capacity, water-binding capacity, and elastic and viscous modulus values than traditional wheat flours, albeit producing weaker gels. Crust flours and their gels are darker than crumb-derived equivalents. The properties exhibited by these wasted bread flours suggest their potential reutilisation as an ingredient in various food products, thereby contributing to the sustainable management of food waste.

In the study entitled “Regionalized Strategies for Food Loss and Waste Management in Spain under a Life Cycle Thinking Approach” by Hoehn et al. [7], food loss and waste (FLW) management strategies in Spain’s 17 regions were assessed for their potential contribution towards a circular carbon economy and compliance with the Paris Agreement targets. Using a Life Cycle Assessment, this study evaluated environmental performance between 2015 and 2040 under five different FLW management scenarios. The results indicated that scenarios employing thermal treatment yield savings in abiotic resources but generate higher greenhouse gas emissions, thereby straining compliance with climate targets. Conversely, those incorporating anaerobic digestion or aerobic composting deliver lower impacts, including impacts on climate change, thus improving compliance by 20–80% relative to current scenarios. This study highlights the importance of region-specific strategies in managing FLW sustainably.

The article entitled “Bacteria and Metabolites: Sustainable Application of Fish Peptones in Nutritive Fermentation Media” by Vázquez et al. [8] explores the use of fish peptones, which were derived from discarded fish species through enzyme proteolysis, as a resource in fermentation processes involving lactic acid bacteria (LAB). The researchers examined the growth and metabolite production of four LAB strains in media formulated with fish peptones. The results indicated that fish peptones supported similar growth rates and metabolite production as a commercially available control broth in 87% of cases. Additionally, an economic assessment revealed that substituting the commercial broth with media containing fish peptones could reduce costs by three- to four-fold. These findings suggest that fish peptones can serve as an effective, economical, and sustainable alternative to commercial peptones, thereby valorising discarded fish biomass and by-products.

In the study entitled “Are Students Really Cautious about Food Waste? Korean Students’ Perception and Understanding of Food Waste”, Islam [9] investigated the perceptions and understandings of food waste among Korean students. This study found significant variance in students’ perceptions across different clusters. “Considerate food wasters” were more informed about food waste and its environmental impacts, and further information on food preservation could potentially enhance their waste reduction practices. However, “unwitting and ruthless food wasters” required more attention, suggesting a need for educational campaigns to raise awareness and influence shopping behaviours to reduce food waste. This research underscores the importance of tailored education to promote sustainable behaviours around food waste among young people.

In the study entitled “From by-product to Unconventional Vegetable: Preliminary Evaluation of Fresh Fava Hulls Highlights Richness in L-Dopa and Low Content of Anti-Nutritional Factor” by Renna et al. [10], faba bean hulls, a typically discarded by-product, were assessed for their potential as a new functional food. The hulls were found to have low levels of vicine, a harmful compound, and high levels of beneficial substances, such as phenols and L-dopa, that are used in treating Parkinson’s disease. Irrespective of the genotype, higher plant density increased pod yield. These findings suggest that these hulls could serve as an unconventional vegetable, particularly beneficial to patients with Parkinson’s disease, thus contributing to sustainable food waste management.

In the article entitled “Spent Coffee Waste as a Potential Media Component for Xylanase Production and Potential Application in Juice Enrichment” by Ravindran et al. [11], spent coffee waste (SCW) is utilised as a carbon source for xylanase production using *Aspergillus niger*. Using a Box–Behnken design, optimal fermentation conditions were determined. Enzyme activity under these conditions was significantly higher than the control, demonstrating the potential of SCW in this process. Furthermore, the application of purified xylanase as a juice enrichment agent for various fruits was examined, further highlighting the possibilities of SCW as a tool for sustainable utilisation and management of food waste.

In the review article entitled “Food Waste Biorefinery: Pathway towards Circular Bioeconomy” by Tsegaye et al. [12], the authors explore the potential of food waste biorefineries as a pathway towards a circular bioeconomy. Biorefineries can use food waste to produce biofuels, chemicals, and bio-based materials, thus greatly reducing environmental impact while creating sustainable resources. The authors highlight the role of waste biorefineries in promoting a green and sustainable economy, thereby contributing to sustainable development goals. This review also emphasises the rising importance of food waste biorefineries due to changing regulations and policies favouring sustainable development. This article provides insight into the current state of food waste biorefineries and their associated products.

Collectively, these articles demonstrate the diverse potential of food waste as a resource for various high-value products, employing innovative and sustainable methodologies. They underscore the importance of tailored, region-specific strategies and circular economy approaches in managing food waste. A common thread across these studies is the practical application of food waste in creating biofuels, high-value food ingredients, and sustainable packaging materials. Despite the promise shown, some contradictions arise regarding the environmental impacts of different food waste management strategies. These studies emphasise the need for a robust methodology in assessing food waste management strategies, as well as increasing public awareness and education about food waste and its sustainable management.

This Special Issue underscores the pressing need and vast potential for sustainable utilisation of food waste. The scientific inquiries presented in this Special Issue offer a promising glimpse into the myriad of possibilities that lie within what is traditionally seen as “waste”. The implications are profound—turning the global food waste problem on its head can lead not only to significant environmental and economic benefits but also to societal advancements through enhanced food security and health benefits.

This body of work further emphasises the importance of interdisciplinary collaboration by involving stakeholders from all corners of society—from researchers and policymakers to industry practitioners and consumers. By continuing research in this field and fostering open dialogue, we can collectively create solutions that both address the food waste problem and foster sustainable development.

I sincerely hope that this Special Issue will inspire and drive further research, catalyse policy shifts, and influence practices within industries. We are at the dawn of a new era where waste can be seen not as a problem but as a valuable resource, a shift that can have transformative impacts on our societies and environments.
